# Glycolytic Switch in Response to Betulinic Acid in Non-Cancer Cells

**DOI:** 10.1371/journal.pone.0115683

**Published:** 2014-12-22

**Authors:** Elke H. Heiss, Matthias P. Kramer, Atanas G. Atanasov, Hortenzia Beres, Daniel Schachner, Verena M. Dirsch

**Affiliations:** Department of Pharmacognosy, University of Vienna, Althanstrasse 14, 1090, Vienna, Austria; University of Mississippi, United States of America

## Abstract

The naturally occurring triterpenoid betulinic acid (BA) shows pronounced polypharmacology ranging from anti-inflammatory to anti-lipogenic activities. Recent evidence suggests that rather diverse cellular signaling events may be attributed to the same common upstream switch in cellular metabolism. In this study we therefore examined the metabolic changes induced by BA (10 µM) administration, with focus on cellular glucose metabolism. We demonstrate that BA elevates the rates of cellular glucose uptake and aerobic glycolysis in mouse embryonic fibroblasts with concomitant reduction of glucose oxidation. Without eliciting signs of obvious cell death BA leads to compromised mitochondrial function, increased expression of mitochondrial uncoupling proteins (UCP) 1 and 2, and liver kinase B1 (LKB1)-dependent activation AMP-activated protein kinase. AMPK activation accounts for the increased glucose uptake and glycolysis which in turn are indispensable for cell viability upon BA treatment. Overall, we show for the first time a significant impact of BA on cellular bioenergetics which may be a central mediator of the pleiotropic actions of BA.

## Introduction

Betulinic acid (3β-3-Hydroxy-lup-20(29)-en-28-oic acid; BA) is a naturally occurring pentacyclic triterpenoid with a multifaceted activity profile. Multiple studies revealed among others anti-viral, anti-proliferative, pro-apoptotic, anti-inflammatory, vasoprotective, as well as anti-diabetic and anti-lipogenic properties for BA and its derivatives both *in vitro* and *in vivo* [1]–[11]. In line with the plethora of reported bioactivities several molecular targets have been proposed including the nuclear factor κB - [12], the sterol regulatory element binding protein -[7], and the endothelial NO synthase pathway [5], the mitochondrial permeability transition pore (MPTP) [13], diacylglycerol acyltransferase [14], the Tgr5 bile acid receptor [6], lipases [15] or protein tyrosine phosphatase 1B [16].

It has recently become more and more appreciated that the metabolic program is not a passive bystander but an active modulator of signal transduction and phenotype of a cell [17]. A change in the metabolic program can influence at once multiple and at first sight unrelated signaling pathways, e.g. by providing or limiting pivotal substrates for anabolism, cytoprotection or posttranslational modifications, and be seen as one central upstream determinant of cellular behavior [18].

Hypothesizing that some of the bioactivities exerted by BA are a consequence of altered bioenergetics we set out to investigate the impact of BA on glucose metabolism.

## Materials and Methods

### Cells, chemicals and antibodies

Wild type (WT) and isogenic AMPKα_1_ -/- mouse embryonic fibroblasts (MEF) and WT and LKB1 -/- MEF were kind gifts from Benoit Viollet, INSERM Paris, France and Reuben Shaw, Scripps Institute, La Jolla, USA, reported in [19] and [20], respectively. Murine 3T3-L1, C2C12, RAW 264.7 cells were from LGC/ATCC (Wesel, Germany). Primary human endothelial cells (HUVEC) were from Lonza (Braine-L'Alleud, Belgium). Betulinic acid (99% purity) was purchased from Biosolutions Halle GmbH (Halle, Germany). Tritium-labeled 2-deoxyglucose (DOG) was provided by NEN (Vienna, Austria). The CellTiterGlo, the CaspaseGlo- and the CytoTox96 non-radioactive cytotoxicity assays came from Promega (Mannheim, Germany). MitoTracker Green and MitoSox Red were purchased from Invitrogen (Vienna, Austria). Special cell culture plates, cartridges, calibrant solution as well as glycolysis and mitochondrial stress test kits were ordered from Seahorse Biosciences. STO609 came from Calbiochem. Primary anti-AMPK (#2532), anti-pAMPK (Thr172) (#2535), anti-pACC (Ser79) (#3661), the anti-LKB1 (#3047) anti-PDHE1 (#2784) as well as the antibodies directed against glycolytic enzymes (glycolysis sampler kit) came from Cell Signaling Technology (Frankfurt am Main, Germany). The anti-GLUT1 and GLUT3 antibodies came from Millipore (Vienna, Austria) (#CBL242; #AB1344), the anti- UCP1 or 2 antibodies were ordered from Abcam (Cambridge, UK) (#10983, 77363), the anti-p-PDHE1 (Ser273) was from Novus Biologicals (Cambridge, UK) (# NB11093479) and the anti-actin antibody was from mpbio (Eschwege, Germany) (#69100). Secondary horse radish peroxidase (HRP)-coupled anti-rabbit and anti-mouse antibodies came from Cell Signaling Technology and mpbio, respectively, and the HRP-anti-goat antibody was from Santa Cruz (Heidelberg, Germany). All other chemicals were from Sigma-Aldrich (Vienna, Austria). All test compounds or inhibitors were dissolved in DMSO, protected from light as far as possible, aliquoted and stored at −20°C. For cell experiments, the final concentration of DMSO was kept constant in all samples and never exceeded 0.3% DMSO.

### Cultivation of cells

Except for HUVEC cells were routinely subcultivated in Dulbecco's modified essential medium (DMEM, 4.5 g/L glucose from Lonza) supplemented with 10% heat inactivated fetal calf serum (Invitrogen) and 2 mM glutamine (Lonza). HUVEC cells were grown in endothelial growth medium (EGM1) and supplements provided by Lonza. For differentiation of 3T3-L1 cells to mature adipocytes and of C2C12 myoblasts to myotubes standard protocols were used as described elsewhere [21], [22]. Cells were routinely tested as mycoplasma-free and kept in culture <11 passages (for primary HUVEC <5).

### Determination of the cellular glucose uptake rate

Determination of the cellular glucose uptake rate was performed as previously described [23]. Briefly, cells were prepared in 12-well plates. After treatment as indicated cells were equilibrated in standard Krebs Ringer Phosphate HEPES (KRPH) buffer containing 0.2% bovine serum albumin (BSA) for 20 minutes. The glucose uptake was initiated by addition of 2-DOG spiked with 2-deoxy-D-(1H3)-glucose (final concentrations 0.1 mM and 0.45 µCi/mL). After 15 min the reaction was stopped by three rapid washes with ice-cold PBS. The glucose uptake rate was determined by liquid scintillation counting (Perkin Elmer, Brunn am Gebirge, Austria) of cell lysates (lysis by 0.05 N NaOH in PBS), normalized to protein content assessed by the Rotiquant™ (Carl Roth, Karlsruhe, Germany) protein assay and uptake time (to obtain incorporated radioactivity per mg protein and minute) and corrected for the non-transporter-mediated glucose uptake (which is not inhibited by co-treatment with cytochalasin B (10 µM) during the uptake procedure). Oligomycin A (2 µM, 4 h) served as positive control for increased basal glucose uptake.

### Assessment of potential cytotoxicity via determination of released lactate dehydrogenase (LDH), ATP levels, caspase cleavage and biomass

MEFs were grown in 96-well plates (seeding density 2.5×10^4^ cells/well). After treatment as indicated we determined the release of LDH (measure for membrane integrity), ATP levels (measure for cell viability) and caspase cleavage (measure for apoptosis induction) with the CytoTox96 Non Radioactive Cytotoxicity Assay, the CellTiterGlo Luminescent Cell Viability Assay and the CaspaseGlo 3/7 Luminescent Assay, respectively. All kits were performed according to the provided protocols. Biomass was stained by pouring off medium and incubating attached cells with crystal violet solution (0.5% (w/v) crystal violet/20% (v/v) MeOH) for 5–10 minutes. After thorough washing steps with tap water (in order to get rid of excess dye) and drying the bound dye was solubilized with an alcoholic citrate solution (0.05 M citrate/50% (v/v) EtOH) and quantified by absorbance readings at 595 nm. Absorbance and luminescence were monitored in a Tecan (Grödig, Austria) Sunrise and GeniosPro plate reader, respectively. Triton (1%), staurosporine (1 µM) or a combination of oligomycin A (2 µM) and DOG (10 mM) served as positive controls for the respective assays.

### Nuclear factor E2-related factor 2 (Nrf2)-dependent reporter gene assay

The Nrf2-dependent reporter gene assay was based on luciferase expression triggered by the activated antioxidant response element (ARE) of murine glutathione-*S*-transferase and performed as previously described [24]. CDDO-IM, a synthetic triterpenoid (100 nM), served as positive control and was kindly provided by Michael Sporn, Geisel School of Medicine at Dartmouth, Hanover, NH, USA.

### Extracellular flux analysis for determination of glycolytic and respiratory capacities

MEF were seeded in appropriate collagen-coated 24-well cell cultures plates (from Seahorse Biosciences; Copenhagen, Denmark) cell density 2.7×10^4^ cells/well). After treatment as indicated cells were kept in serum free medium (DMEM plus 2 mM glutamine, 0 mM glucose, 0 (glycolysis stress test) or 2 (mitochondrial stress test) mM pyruvate, pH 7.35–7.40) at 37°C and ambient CO_2_ for one hour before they were subjected to glycolysis (readout: extracellular acidification rate (ECAR) in mpH/min) and mitochondrial (readout: oxygen consumption rate (OCR) in pmole O_2_/min) stress tests. Appropriate test kits came from Seahorse Biosciences, were performed according to the manufacturers' instructions and analyzed on a Seahorse 24XF^e^ extracellular flux analyzer and Wave software (www.seahorsebio.com). Optimized inhibitor concentrations for MEF were 2 µM oligomycin A, 1.5 µM carbonylcyanid-p-trifluoromethoxyphenylhydrazon (FCCP), 1 µM rotenone A, 1 µM antimycin A, and 100 mM 2-DOG. Normalization to cell mass was routinely performed by crystal violet staining after the analysis in order to account for potential differences in cell number.

### Determination of the extracellular lactate as marker for glycolysis

MEFs were prepared in 24-well plates. After treatment as indicated cells were washed with PBS and then incubated with standard Krebs Ringer Phosphate HEPES (KRPH) buffer supplemented with 0.2% BSA and 10 mM glucose for 2 hrs. Then supernatants were analyzed for their lactate content via an enzyme-coupled fluorescence assay, and cells were lysed and their protein content was determined. Briefly, one volume supernatant (usually diluted 1∶20) was mixed with one volume assay buffer (KRP buffer with 10 µM Amplex Red, 1 U/mL lactate oxidase and 2.5 U/mL horseradish peroxidase), incubated for 10 minutes and then read in a flourimeter at an excitation wavelength of 535 nm and emission wavelength of 590 nm. Parallel monitoring of solutions with known concentrations of lactate facilitated a final readout of mol lactate/g protein* min.

### Flow-cytometric determination of mitochondrial content and mitochondrial reactive oxygen species (ROS) production

MEF were grown in 12- or 6- well plates (seeding density 1.5×10^4^ or 3×10^4^ cells per well) and treated as indicated. For determination of the mitochondrial content cells were stained with 50 nM MitoTracker Green (Invitrogen) for 20 minutes and then analyzed in the green channel (FL1, ex 488 nm; em 530/30 nm) of the FACS Calibur (BD Biosciences, Schwechat, Austria). The arbitrary mean of green fluorescence was taken as measure for the mitochondrial content after correction for autofluorescence [25], [26]. Valinomycin served as control for the potential-independence of the MitoTracker Green signal. For determination of mitochondrial ROS production cells were incubated with 5 µM MitoSox Red (Invitrogen) for 10 min and then analyzed for their autofluorescence-corrected red fluorescence (FL2 channel, ex 488 nm; em 585/42 nm) in the flow cytometer. The arbitrary mean of red fluorescence was taken as readout for produced mitochondrial ROS. Antimycin A (2 µM) served as positive control in this assay.

### Protein extraction, SDS-polyacrylamide electrophoresis and immunoblot analysis

MEFs were prepared in 6-well plates and treated as indicated. Immunoblot analysis including protein extraction, gel run, transfer, immunodetection and densitometric evaluation were performed as described elsewhere [23]. For detection of GLUT1 and 3 samples were not boiled prior to electrophoresis in order to prevent aggregation and precipitation. For detection of proteins of similar size (e.g. phosphorylated form versus total protein) two identical membranes out of the same protein extracts were prepared and evaluated in order to avoid potential artefacts or ambiguous signals due to incomplete stripping.

### Statistics

For statistical analyses experiments were performed at least three times (n≥3; independent experiments (biological replicates)). Bar graphs depict mean + SD. Two groups were compared using Student's t test, more groups were analyzed by one-or two-way ANOVA depending on the number of variables in the investigated data sets, followed by Dunnett's or Bonferroni's post hoc test. All statistical analyses were done with GraphPad Prism. Differences with p values <0.05 were considered as significant and are designated with *.

## Results

### BA increases cellular glucose uptake

**Figure 1:**
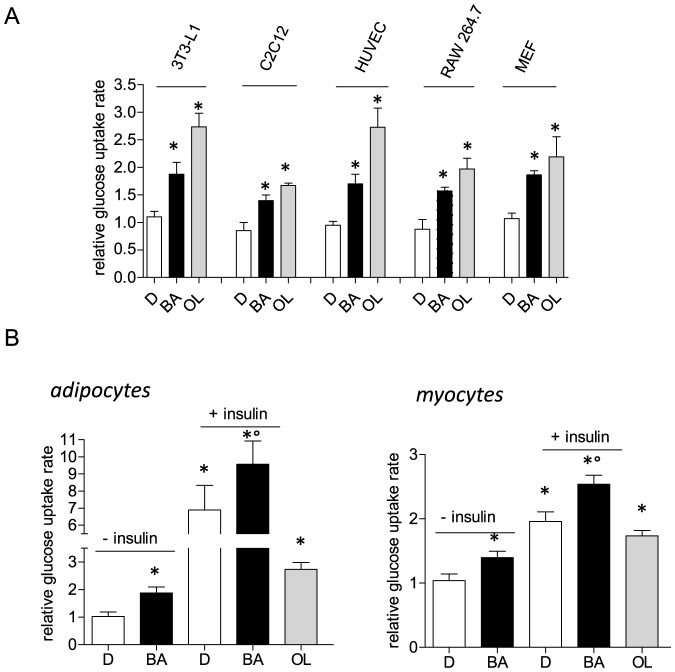
Cellular glucose uptake rate upon exposure to BA. (A) Different cell types (differentiated adipocytes (3T3-L1), differentiated myotubes (C2C12), endothelial cells (HUVEC), macrophages (RAW264.7) and murine embryonic fibroblasts (MEF)) were treated with 10 µM BA (+) or 0.1% DMSO (−) for 16 h before their cellular glucose uptake rate was determined as described. Oligomycin A (OL, 2 µM for 4 h) served as positive control for increased basal glucose uptake. The bar graph depicts glucose uptake rates relative to the mean of the respective DMSO control (n = 3 (i.e. three independent experiments, each in triplicate); * p<0.05 vs DMSO ctrl, ANOVA). (B) Differentiated adipocytes (left) and myocytes (right) were treated with BA (10 µM) for 16 h prior to serum starvation and insulin stimulation (15 min; 3T3-L1: 15 nM insulin; C2C12: 100 nM insulin). Then cellular glucose uptake was determined. Bar graphs depict glucose uptake rates relative to the mean of the respective unstimulated vehicle control. (n = 3 (i.e three independent experiments, each in triplicate); mean + SD; * p<0.05 vs unstimulated DMSO ctrl, ° p<0.05 vs insulin-stimulated DMSO ctrl, two-way ANOVA, Bonferroni). Oligomycin A (OL, 2 µM for 4 h) served as positive control for increased basal glucose uptake.

First we assessed the cellular glucose uptake rate after treatment with BA in different cell types, including primary human endothelial cells (HUVEC), immortalized murine macrophages (RAW264.7), differentiated mouse myo (C2C12)- and adipocytes (3T3-L1) as well as murine embryonic fibroblasts (MEF). We consistently observed an increased basal glucose uptake into all cell types by approximately 50 to 100% (Fig. 1A) with BA at a concentration at 10 µM (quasi-optimal concentration for all used cell lines as determined by concentration-response experiments; S1 Fig.). Oligomycin A, an inhibitor of the mitochondrial ATP synthase, was used as positive control. These findings suggested a general and cell-type independent increase of glucose incorporation by BA. Addition of saturating concentrations of insulin to differentiated adipocytes and myocytes, two major insulin-sensitive glucose disposing cell types, led to an increased cellular glucose uptake on top of the BA effect (Fig. 1B) pointing to an insulin-independent action of BA.

### BA does not induce cell death in MEF

**Figure 2:**
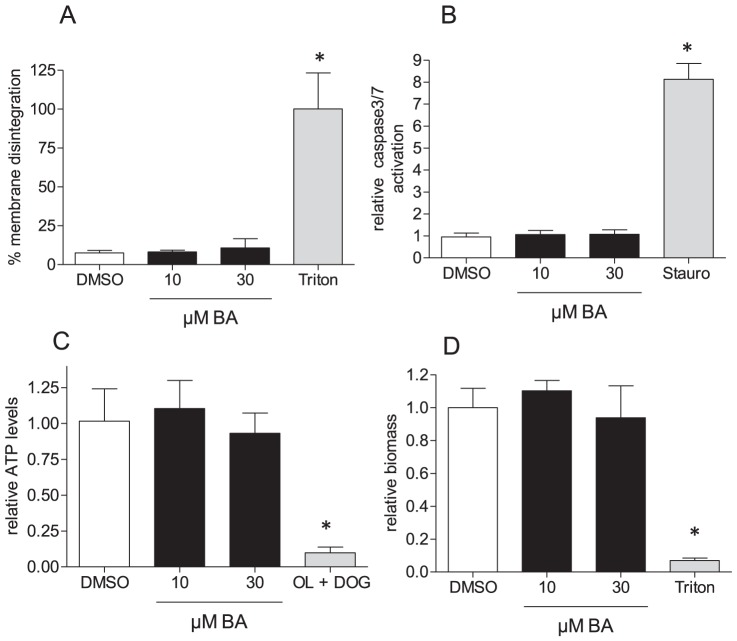
Cytotoxicity of BA in MEF. MEF were treated with BA (10 µM and 30 µM) for 48 h before they were subjected to determination of membrane integrity (% LDH release) (A), potential of proapoptotic events (activation of caspase 3/7) (B), of ATP levels (C) and biomass (D). Bar graphs depict compilation of three independent experiments (expressed as fold of the DMSO mean value in B, C, and D), each in quadruplicate (mean + SD, * p<0.05, ANOVA, Dunnett's post test versus DMSO ctrl). Staurosporine (Stauro, 1 µM for 6 h), triton (1% for 1 h) or a combination of oligomycin A (OL; 2 µM) and DOG (10 mM; 5 h) served as positive controls in the assays.

As increased glucose uptake may indicate cellular stress and cytotoxicity, and BA has been shown to exert pro-apoptotic effects in various (cancer) cell types, we next determined whether the observed elevated glucose uptake is associated with subsequent cell death. We treated MEF, the cell type selected for all further studies, with 10 µM BA for 48 h and determined the relative release of lactate dehydrogenase (LDH; ratio of extracellular and total) as readout for cell death and membrane disintegration (Fig. 2A), activation of caspases as readout for apoptosis induction (Fig. 2B), ATP levels as sign of general cell viability (Fig. 2C) and binding of crystal violet as simple biomass stain (Fig. 2D). BA at 10 µM and 30 µM did not induce any significant changes compared to DMSO-treated control cells. Thus, the observed increased glucose uptake upon BA exposure is unlikely due to imminent cell death. Furthermore, BA did not trigger activation of the transcription factor nuclear factor E2 related factor 2 (Nrf2) as shown in an Nrf2-dependent reporter gene assay. Nrf2 activation is well known as indicator for a cellular stress response upon exposure to xenobiotics (S2 Fig.). The lack of toxicity up to concentrations of 30 µM could also be confirmed for endothelial cells, adipocytes and myocytes (S3 Fig.) and supports the general notion of the cancer-selective toxicity of BA.

### BA elevates aerobic glycolysis

**Figure 3:**
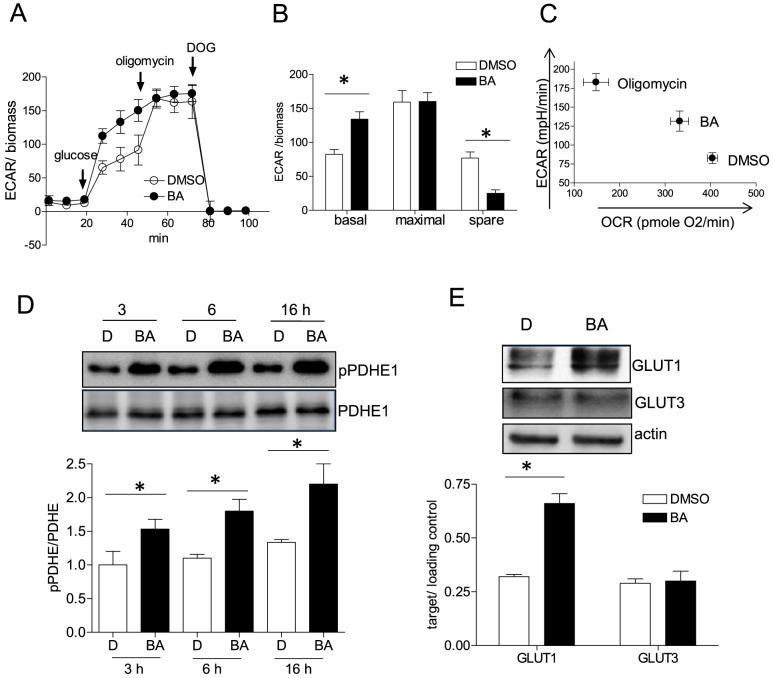
Rate of aerobic glycolysis in the presence of BA. MEF were treated with 10 µM BA or DMSO (0.1%) for 16 h before they were subjected to a glycolysis stress test as described under “Materials and Methods”. In (A) the extracellular acidification rate (ECAR) is depicted upon exposing cells successively to glucose (10 mM), oligomycin A (2 µM) and deoxyglucose (DOG, 100 mM) (mean + SD; compiled raw data from three independent experiments with four technical replicates each). In (B) those data are analyzed in terms of basal (glycolytic ECAR after glucose addition), maximal (glycolytic ECAR after oligomycin) and spare (maximal minus basal activity) glycolytic activity (mean + SD,* p<0.05, t-test, DMSO vs. BA). Quantitation is based on the value at the final time point of each treatment condition. In (C) values of OCR and ECAR (of DMSO and BA-treated cells (16 h)) after addition of glucose were plotted against each other to visualize the shift from glucose oxidation to glycolysis. The observed shift upon addition of oligomycin A is added to the plot as a reference. (D) MEF were treated with DMSO (0.1%, D) and 10 µM BA for the indicated periods of time before total cell lysates were subjected to immunoblot analyses for pPDHE1 (Ser273) and total PDHE1 (molecular weight 43 kDa). Representative blots out of three experiments are shown. The graph below depicts compiled densitometric values of pPDHE/PDHE (n = 3; mean + SD; *p<0.05; t-test; DMSO vs BA at each time point). (E) MEF were treated with 10 µM BA or DMSO (0.1%) for 16 h before they were subjected to immunoblot analysis for GLUT1 (∼50 kDa), GLUT3 (∼55 kDa) and actin (42 kDa). Representative blots out of three independent experiments are shown. The graph below depicts compiled densitometric values of GLUT1/actin and GLUT3/actin, respectively (n = 3; mean + SD; *p<0.05; t-test; DMSO vs BA at each time point).

Next we were interested in the metabolic fate of the ingested glucose. The glycolytic rate was investigated by using extracellular flux analysis. We observed that BA-treated cells showed a significantly higher extracellular acidification rate (ECAR) upon addition of glucose than their vehicle-treated counterparts (Fig. 3A and B). In a complementary assay determining extracellular lactate levels we consistently monitored elevated lactate production by BA-treated cells (S4 Fig.). These findings indicate a higher glycolytic rate in BA- treated cells and excluded an influence of a putative membranous V-ATPase to the observed acidification. The maximal glycolytic capacity (ECAR after oligomycin addition and inhibition of mitochondrial ATP production) is comparable between DMSO- and BA-treated cells. Consequently, BA-treated cells possess a significantly decreased glycolytic spare capacity (ΔECAR_maximal_- ECAR_basal_)(Fig. 3B). These data show that BA drives cells towards full exploitation of their glycolytic potential in disfavor of glucose oxidation (Fig. 3C). An observed increased phosphorylation of pyruvate dehydrogenase E1 (PDHE1) further alluded to a glycolytic switch upon BA treatment. Phosphorylation renders PDHE1 less active and interferes with oxidative decarboxylation of pyruvate to acetyl-CoA favoring pyruvate reduction to lactate (Fig. 3D). However, further experiments will be needed to unambiguously assess to what extent PDHE phosphorylation contributes the near maximal glycolysis in BA-treated cells. The expression level of several investigated glycolytic enzymes was not changed (S5 Fig.) which, however, does not exclude an altered enzymatic activity due to covalent or allosteric modification. The expression of glucose transporter GLUT1 was elevated upon BA treatment for 16 h whereas the levels of GLUT3 were not altered (Fig. 3E). GLUT2/4 were not detectable in our MEF.

### BA impairs mitochondrial function

**Figure 4:**
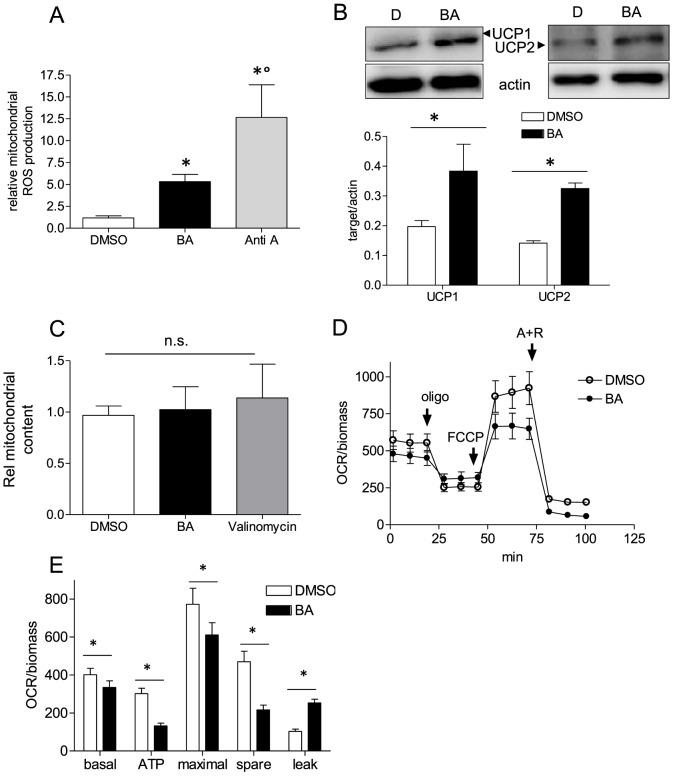
Mitochondrial function in the presence of BA. (A) MEF were treated with 10 µM BA or 0.1% DMSO for 16 h before they were subjected to flow cytometric analysis of the mitochondrial ROS production with the use of MitoSox Red and antimycin A (2 µM, 1 h) as positive control. (n = 3 (each in duplicate or triplicate); mean + SD,* p<0.05, t-test). (B) MEF were treated with DMSO or 10 µM BA for 16 h before total cell lysates were subjected to western blot analysis for UCP1, UCP2 (band at ∼25–35 kDa) and actin (42 kDa) as loading control. Representative blots of three independent experiments are depicted. The graph below depicts compiled densitometric values of UCP1/actin and UCP2/actin, respectively (n = 3; mean + SD; *p<0.05; t-test; DMSO vs BA). (C) MEF were treated with 10 µM BA or 0.1% DMSO for 16 h before they were subjected to flow cytometric analysis of the mitochondrial content by using MitoTracker Green. Valinomycin (200 nM, 16 h), a known disruptor of the mitochondrial membrane potential served as control for the potential-independent signal of MitoTracker Green. MEF were treated with 10 µM BA or DMSO (0.1%) for 16 h before they were subjected to a mitochondrial stress test as described under Materials. In (D) the mean oxygen consumption rate (OCR) of three independent experiments is depicted upon exposing cells successively to oligomycin (2 µM), FCCP (1.5 µM) and antimycin A + rotenone (A+R; 1+1 µM). In (E) those data are analyzed in terms of oxygen consumption rate under basal conditions, oxygen consumption for ATP synthesis, maximal respiratory rate, spare respiratory capacity and proton leak (mean + SD,* p<0.05, t-test, DMSO vs. BA).

As increased aerobic glycolysis may compensate for an impaired ATP production by oxidative phosphorylation we next examined mitochondrial function after BA treatment. We observed increased production of mitochondrial ROS (Fig. 4A) and elevated expression of uncoupling proteins UCP1 and UCP2 in BA-treated MEF (Fig. 4B). The total mitochondrial content remained unaffected (Fig. 4C). Altered mitochondrial function was confirmed in a mitochondrial stress test and extracellular flux analysis. Cells displayed a slightly reduced basal oxygen consumption rate (OCR), a reduced coupling of oxygen consumption to mitochondrial ATP production (Δ(OCR_basal_-OCR_oligomycin_), diminished maximal respiration (OCR after dissipation of the proton gradient by FCCP), a reduced respiratory spare capacity (Δ(OCR_maximal_-OCR_basal_)), as well as an increased proton leak (Δ(OCR_oligomycin_ – OCR_A+R_)) when treated with BA for 16 h (Figs. 4E and F). These data indicate that treatment with BA interferes with mitochondrial function, however, mildly as no decrease in cell viability is triggered by BA (see Fig. 2). Time course experiments revealed that uncoupled respiration (evident in the decrease of OCR used for ATP production in S6A Fig.) correlated with the time-dependent induction of UCPs (S6B Fig.). However, the issues whether UCP induction accounts for the observed uncoupling or whether BA itself as rather hydrophobic molecule with a pKa value of 5.5 is a weak uncoupler need further investigation. Notably, upon short incubations with BA (1–3 h) basal OCR tends to be increased compared to DMSO control cells as expected for uncoupled mitochondrial respiration (S6A Fig.). The later reduction of OCR seen in BA-treated cells may be due to still elusive adaptive or additional mechanisms triggered by the triterpenoid such as dissipation of the mitochondrial membrane potential over time or an impaired electron flow through the respiratory chain.

### BA activates AMP-activated protein kinase (AMPK)

**Figure 5:**
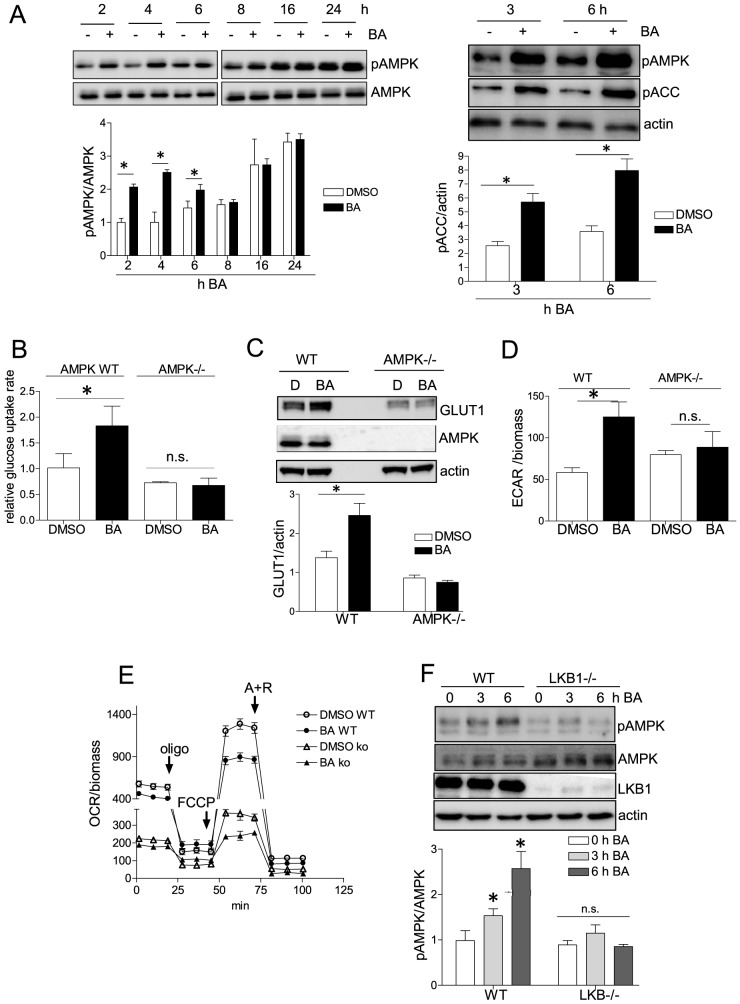
The role of AMPK and LKB-1 for the BA-induced glycolytic switch. (A) MEF were treated with DMSO (0.1%) or BA (10 µM) for the indicated periods of time. Total cell lysates were subjected to immunoblot analysis for pAMPK (Thr172) and total AMPK (60 kDa) (left panel) or pAMPK (Thr172), pACC (Ser79) (∼245 kDa) and actin (42 kDa) (right panel). Representative blots out of three independent experiments are depicted. The graphs below depict compiled densitometric values of pAMPK/AMPK or pACC/actin, respectively (n = 3; mean + SD; *p<0.05; t-test; DMSO vs BA at each time point). WT and AMPK -/- MEF were treated with DMSO or BA (10 µM) for 16 h. Then the cellular glucose uptake rates (B) were assessed (n = 3 (in triplicate); mean + SD,* p<0.05, ANOVA, Dunnett's post-test vs DMSO ctrl), as well as the expression levels of GLUT1 (50 kDa), AMPK (60 kDa) and actin (42 kDa) by western blot analysis (C). One representative blot is depicted of three independent experiments. The graph below depicts compiled densitometric values of GLUT1/actin, respectively (n = 3; mean + SD; *p<0.05; t-test; DMSO vs BA). In (D) cells were subjected to determination of the extracellular acidification rate (ECAR) as described in Fig. 3. Representative results of three independent experiments (with three technical replicates each) are shown (mean + SD,* p<0.05, t-test). (E) WT and AMPK -/- MEF were treated with DMSO (0.1%) or BA (10 µM) for 16 h before they were subjected to a mitochondrial stress test and extracellular flux analysis. Compiled data of three independent experiments are depicted (mean + SD). (F) WT and LKB1-/- MEF were treated with BA (10 µM) for the indicated periods of time before total cell lysates were subjected to immunoblot analysis for pAMPK (Thr172), AMPK (60 kDa), LKB1(∼50 kDa) and actin (42 kDa). Representative blots out of three independent experiments are depicted. The graph below depicts compiled densitometric values of pAMPK/AMPK, respectively (n = 3; mean + SD; *p<0.05; ANOVA, Dunnett (vs 0 h treatment with BA).

Reduced mitochondrial function has been linked with activation of AMPK (reviewed in [27]), one central metabolic master hub. AMPK activation can furthermore explain a compensatory increased glucose uptake and glycolytic rate [28], [29]. We therefore investigated whether BA leads to activated AMPK. Using immunoblot analysis we observed a transient boost of AMPK phosphorylation at threonine 172, indicative of elevated AMPK activity, in BA- treated versus control cells (Fig. 5A). AMPK activation was further corroborated by increased phosphorylation of acetyl-CoA carboxylase (ACC) at serine 79, a common downstream target of AMPK. The use of isogenic WT MEF and AMPK knockout counterparts revealed that increased glucose uptake, increased expression of the glucose transporter GLUT1 and elevated glucose metabolism via glycolysis were dependent on the presence of AMPK (Fig. 5B, C, D). However, BA induced a comparable reduction of mitochondrial function (e.g. reduction of maximal respiration by approximately 40% compared to DMSO control) in both WT and AMPK -/- MEF. This finding placed mitochondrial dysfunction upstream of AMPK activation (Fig. 5E) being in line with the observed activation of AMPK, induction of GLUT1, an elevated glucose uptake and an increased glycolytic rate upon mild FCCP-imposed uncoupling (S7 Fig.). Of note, AMPK -/- cells showed a generally lower oxygen consumption rate than WT MEF probably partly due to their reduced number of mitochondria (S8 Fig.). Comparing WT and cells deficient in liver kinase B1 (LKB1), the AMPK kinase responsive to an altered AMP/ATP ratio [30], suggested LKB1 as the major AMPK kinase involved: LKB1 -/- cells displayed diminished AMPK phosphorylation in upon BA treatment (Fig. 5F).

These data showed that BA mildly impaired mitochondrial function, triggered AMPK activation via LKB1 which in turn elicited increased glucose intake and shifted cellular glucose metabolism from oxidation to aerobic glycolysis.

### BA renders cells addicted to glucose

**Figure 6:**
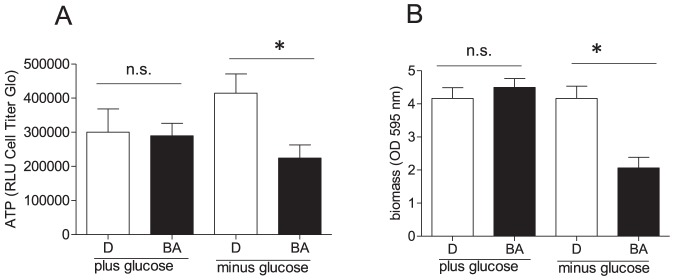
Glucose-addiction in the presence of BA. MEF were treated with DMSO (0.1%, D) or BA (10 µM) in DMEM (containing 1% serum, glutamine (2 mM) and pyruvate (2 mM)) in the presence (25 mM) and absence of glucose (instead 25 mM mannitol as osmotic balance) for 48 h before ATP levels (A) and biomass (B) were assessed. Bar graphs depict compiled data of three experiments in quadruplicate. (mean + SD, * p<0.05, t-test DMSO vs BA).

Intrigued by the finding that BA drives cells into enhanced glycolytic activity we next tested whether BA rendered cells glucose addicted. For this we assessed cell viability (ATP levels, biomass) of MEF treated with vehicle or BA in the absence and presence of glucose for 48 h. For glucose-deprived cells we added mannitol as osmotic balance. DMSO-treated control cells coped well with glucose depletion as evident by unaltered biomass and ATP levels compared to the “plus glucose” condition. In the absence of glucose those cells even showed a reproducible trend to elevated ATP levels which may be explained by forced mitochondrial oxidation of substrates (e.g. fatty acids contained in the serum) with higher ATP yield than glucose. BA in the presence of glucose did not affect our chosen readouts of cell viability either (consistent with Fig. 2). Unaltered ATP levels despite impaired mitochondrial respiration in BA-treated cells can be explained by compensation via their increased glycolytic rate. However, treatment with BA under glucose free conditions led to a significant and major drop in total cellular ATP levels and biomass (Fig. 6A and B) underlining glucose addiction of BA-treated cells.

## Discussion

We investigated the impact of the lupane-type triterpenoid BA on cellular glucose metabolism and bioenergetics in MEF. We show here that BA (i) decreases the oxidative capacity, (ii) elicits elevated UCP expression, (iii) triggers LKB1-dependent AMPK activation without causing cell death, and that (iii) activated AMPK accounts for the increased uptake and metabolization of glucose via glycolysis after BA treatment. (iv) Moreover, BA apparently renders cells addicted to glucose.

Of note, BA induces a metabolic reprogramming that is highly reminiscent of the Warburg effect (aerobic glycolysis favored over mitochondrial respiration) in tumor cells [31]. BA also triggers apoptosis rather selectively in cancer cells [32]. Further studies focusing on BA as anti-cancer agent should investigate whether the BA-driven shift in cellular metabolism also occurs in cancer cells and is involved in the cancer cell-selective proapoptotic effect. Preliminary studies from our lab indicate that BA indeed further elevates the already high rate of glycolysis in HT116 colon cancer cells. One may therefore reason that incubation with BA drives cancer cells which already heavily rely on glycolysis for energy production into absolute addiction to glycolysis (“super-Warburg effect”) and possibly over the limit of glucose supply. Alternatively, the activation of the LKB1-AMPK axis by BA may act as the metabolic tumor suppressor as comprehensively reviewed in [33], [34]. Interestingly, reduced glucose oxidation was recently observed in prostate cancer cells exposed to metformin, an accepted AMPK activator. The authors of that study further revealed increased dependency on reductive glutamine metabolism and a consequent synergy between metformin and interference with glutamine metabolism, an issue which may also apply for the anti-cancer action of BA [35].

An influence of pentacyclic triterpenoids, including the lupane-type betulin, BA or dimethylaminopyridine derivatives of BA on mitochondrial function and respiration has already been reported [13], [36]–[38]. Existing studies mainly focused either on cancer cells/tumor models or used higher concentrations of BA (>15 µM) with the ultimate goal of mitochondrial permeability transition pore (MPTP) opening, cytochrome c release and apoptosis induction [13], [39]. Whether the MPTP is involved in our observations regarding metabolism and mitochondrial function in BA-treated MEF is not entirely resolved yet. On the one hand, involvement is conceivable because the MPTP complex is associated with the glycolytic enzyme hexokinase II (HKII) at the outer mitochondrial membrane [40] and MPTP opening was causally linked with HK mitochondrial binding and activity [41], [42]. On the other hand, data from our lab indicate that co-treatment with BA and cyclosporine A (known to overcome the MPTP-mediated effect of BA [38], [43]) rather accentuates than reverses the influence of BA on mitochondrial function and basal glycolytic activity (S9 Fig.). BA leads to up-regulation of both UCP1 and UCP2 in MEF. MEFs provide an excellent model system for cell signaling studies due to their easy cultivation and the availability of multiple isogenic WT and knockout lines. Nonetheless future studies are warranted to confirm increased UCP expression also in other differentiated cells types, especially of UCP1 which is predominantly expressed in brown adipocytes and mediates their thermogenesis [44], [45], and to decipher the molecular mechanism behind UCP induction. Of note, ursolic acid, another triterpenic acid, has been shown to lead to mitochondrial uncoupling as well [46].

Activation of AMPK has recently been associated with the BA-mediated inhibition of gluconeogenesis and non-alcoholic fatty liver disease [7], [47]. The authors of those studies placed calcium-calmodulin dependent kinase kinase (CaMKK)β upstream of AMPK activation by BA. We show an alternative mechanism of AMPK activation upon BA treatment, namely via LKB1. In our experiments the CaMKK inhibitor STO609 cannot overcome AMPK activation (S10 Fig.). However, cells lacking LKB1, the AMP/ATP sensitive AMPK upstream kinase, fail to show increased AMPK phosphorylation upon exposure to BA (see Fig. 5F). These data suggest a transiently (until increased glycolysis compensates for the reduced mitochondrial ATP production) increased AMP/ATP ratio rather than calcium as trigger for AMPK activation in our setting.

Induction of mild mitochondrial uncoupling and AMPK activation may directly or indirectly contribute to multiple bioactivities of BA. Next to the already mentioned anti-cancer effect AMPK activation has been linked to reduced inflammation, to increased endothelial function, to increased fatty acid oxidation, or reduced lipogenesis [48]–[52] which mirrors many of the reported bioactivities of BA. Whether and to what extent AMPK activation by BA actually takes its share to achieve these activities still needs to be clarified in future investigations.

Overall, we have shown a hitherto unknown action of BA on mitochondrial function, the LKB1/AMPK axis and cellular glucose metabolism. With this study we may have initiated a metabolism-driven understanding of the pleiotropic actions of BA which is possibly applicable also for other molecules with pronounced polypharmacology.

## Supporting Information

S1 Fig BA concentration-dependently increases the glucose uptake rate in different non-malignant cell types. Differentiated 3T3-L1 adipocytes, C2C12 myocytes, endothelial cells (HUVEC), macrophages (RAW264.7) and murine embryonic fibroblasts (MEF) were treated over night with rising concentrations of BA (0, 5, 10, and 20 µM) before their glucose uptake rates were determined as described. Compiled data of two independent experiments are depicted.
(EPS)

S2 Fig BA does not trigger the cellular stress response. CHO-ARE Luc cells were treated with the indicated concentrations of BA or CDDO-IM (100 nM, positive control) for 16 h before Nrf2-dependent luciferase induction was assessed in a Tecan GeniosPro luminescence reader. Bar graph depicts fold induction compiled from three independent experiments with four technical replicates each (mean + SD,* p<0.05, ANOVA, Dunnett's post test vs DMSO ctrl).
(EPS)

S3 Fig BA at 30 µM does not elicit membrane disintegration in myo-, adipocytes or endothelial cells. Myocytes (differentiated C2C12), adipocytes (differentiated 3T3-L1) and endothelial cells (HUVEC) were treated with BA (30 µM) for 48 h before they were subjected to determination of membrane integrity (% LDH release). Triton (1% for 1 h) served as positive control in the assay. Bar graph depicts compiled data of three independent experiments (mean + SD; * p<0.05 vs vehicle-treated control cells).
(EPS)

S4 Fig BA treatment leads to elevated release of lactate. MEF were treated with BA (10 µM) for 16 h before their supernatant was assessed for extracellular lactate as described. Bar graph depicts compiled data of three independent experiments (mean + SD; * p<0.05 vs vehicle-treated control cells). Oligomycin (Oligo, 2 µM; 4 h treatment) served as positive control.
(EPS)

S5 Fig BA does not alter expression level of glycolytic enzymes. MEF were treated with BA (10 µM) for 16 h before total cell lysates were subjected to immunoblot analysis for hexokinase 1, phosphofructokinase (PFK)1, aldolase, glycerine aldehyde phosphate dehydrogenase (GAPDH), phosphoglycerate mutase (PGAM), enolase, pyruvate kinase and actin as loading control. Representative blots of two independent experiments are depicted.
(EPS)

S6 Fig Time dependency of BA-induced mitochondrial dysfunction and UCP induction. MEF were treated with the indicated periods of time with BA (10 µM) before they were subjected to (A) a mitochondrial stress test via extracellular flux analysis (compiled data from two independent experiments, mean + SD) and (B) immunoblot analysis for UCP1, UCP2 and actin. Representative blots out of two independent experiments and compiled densitometric data are depicted.
(EPS)

S7 Fig Mild mitochondrial uncoupling by FCCP elicits AMPK activation in an LKB1-dependent manner and an increase in GLUT1 expression, glucose uptake and basal glycolytic rate. (A) WT and LKB-/- MEF were treated with 30 and 100 nM FCCP (concentrations inducing mild uncoupling and an increased glycolytic rate comparable to BA treatment, see D) for 2 h before total cell lysates were immunoblotted for pAMPK and AMPK. Representative blots out of two independent experiments are depicted. (B) WT MEF were treated with 30 and 100 nM FCCP for 16 h before their GLUT1 expression was determined by immunoblot. Representative blots out of two independent experiments are depicted. (C) WT MEF were treated with 30 and 100 nM FCCP for 16 h before their glucose uptake rates were determined in three independent experiments. Bar graph depicts compiled data (mean + SD; * p<0.05). (D) WT MEF were treated with BA (10 µM) and FCCP (30 and 100 nM) for 16 h before they were subjected to a determination of their basal glycolytic rate by extracellular flux analysis. Graph depicts compiled raw data from three independent experiments with at least three technical replicates (mean+ SD; * p<0.05)
(EPS)

S8 Fig AMPK-/- MEF show a reduced mitochondrial content compared to their WT counterparts. WT and AMPK-/- MEF (500,000 cells each) were stained for their mitochondrial content using MitoTracker Green dye and flow cytometric analysis. Bar graph depicts compiled data (arbitrary fluorescence units) of three independent experiments. (mean + SD, * p<0.05; t-test).
(EPS)

S9 Fig Cyclosporine A does not counteract the BA-induced changes in OXPHOS and glycolytic rate. MEF were treated with BA (10 µM) and cyclosporine A (1 and 10 µM) as indicated for 16 h before they were subjected to mitochondrial and glycolytic stress tests and extracellular flux analysis. Bar graphs depict compiled data from two independent experiments.
(EPS)

S10 Fig The CaMKK inhibitor STO609 does not counteract BA-induced AMPK activation. MEF were treated with DMSO (0.1%) or BA (10 µM) for 2 hrs in the presence or absence of STO609 (10 µM) before their lysates were subjected to immunoblot analysis for pAMPK and AMPK. Representative blots out of three independent experiments with consistent results are depicted as well as the compiled densitometric data of those experiments (mean + SD, * p<0.05).
(EPS)
